# Intraoperative and pathological findings of intramedullary amputation neuroma associated with spinal ependymoma

**DOI:** 10.1007/s10014-012-0125-x

**Published:** 2012-11-28

**Authors:** Hidetaka Arishima, Hiroaki Takeuchi, Kenzo Tsunetoshi, Toshiaki Kodera, Ryuhei Kitai, Ken-ichiro Kikuta

**Affiliations:** Department of Neurosurgery, University of Fukui, 23-3, Matsuokashimoaizuki, Eiheiji-cho, Yoshida-gun, Fukui, 910-1193 Japan

**Keywords:** Ependymoma, Intramedullary amputation neuroma, Spinal cord

## Abstract

Amputation neuromas typically arise in injured peripheral nerves; rarely, however, they arise in the spinal cord. We report a rare case of intramedullary amputation neuroma associated with ependymoma in the cervical spinal cord. A 73-year-old woman presented with a 5-year history of progressive gait disturbance. Neurological examination revealed complete motor deficit of her hands and legs. Magnetic resonance imaging of the cervical spine revealed an enhancing mass within the spinal cord at the C6/7 level. The patient underwent C5–C7 laminectomy surgery. During resection of the spinal tumor, we found a whitish string resembling an aberrant nerve root or schwannoma with adhesion to the tumor on the ventral side of the spinal cord. After resecting the tumor, the surgical specimen was cut and separated into a soft greyish tumor (spinal tumor) and the tough whitish string. Histopathological and immunohistochemical examination revealed the former was a spinal ependymoma and the latter was a neuroma. An intramedullary amputation neuroma associated with a spinal ependymoma is rare, and this is the first known case in which intraoprerative findings were clearly shown. Neurosurgeons should be aware that spinal ependymomas might coexist with neuromas.

## Introduction

Amputation neuromas are non-neoplasm tangles consisting of myelinated axons and Schwann cells that sprout from the end of an injured nerve. They typically arise in the peripheral nerves; rarely, however, they arise in the central nervous system, from the spinal cord or brain stem. For example, most of those previously described were discovered in association with or without a history of trauma [1–9] and disruptive disorders, for example demyelinating disease [10, 11], syringomyelia [10], spinal tumors [10, 12], and other spinal cord or brain disease [10, 13–15]. Most small intramedullary neuromas were discovered by microscopy at autopsy [2, 4, 6, 7, 9, 10, 12, 13, 15, 16]. They were usually tiny and sometimes described as microneuromas [6, 12].

Although microscopic examinations and findings of neuromas in the spinal cord have been reported [2, 4, 7, 9–13], there have been no previous reports of macroscopic and operative findings of these lesions. We report a rare case of amputation neuroma with spinal ependymoma, report the intraoperative and pathological findings, and discuss the pathogenesis.

## Case report

### History

A 73-year-old woman presenting with a 5-year history of progressive gait disturbance was referred to our hospital. She had no past history of trauma or particular disease.

## Examination

Neurological and physical examination revealed complete motor deficit of the hands and legs with severe muscle atrophy. She had apparent sensory disturbance of her hands, trunk, and legs. She could not stand up by herself.

**Fig. 1:**
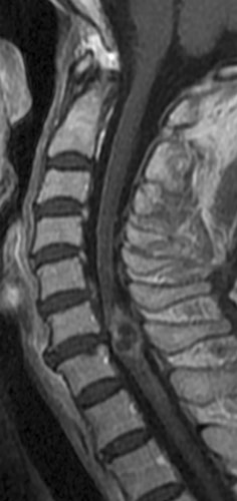
Sagittal T1-weighted magnetic resonance (MR) image with gadolinium revealing an intramedullary lesion with peripheral enhancement at the C6/7 level

Magnetic resonance (MR) imaging of the cervical spine revealed an intramedullary lesion as a hypointense mass on T1-weighted images and a hyperintense mass on T2-weighted images at the C6/7 level. T2*-weighted images detected intramedullary microhemorrhage around the mass. After gadolinium injection, peripheral enhancement of the intramedullary lesion was visualized (Fig. 1).

## Operation

**Fig. 2:**
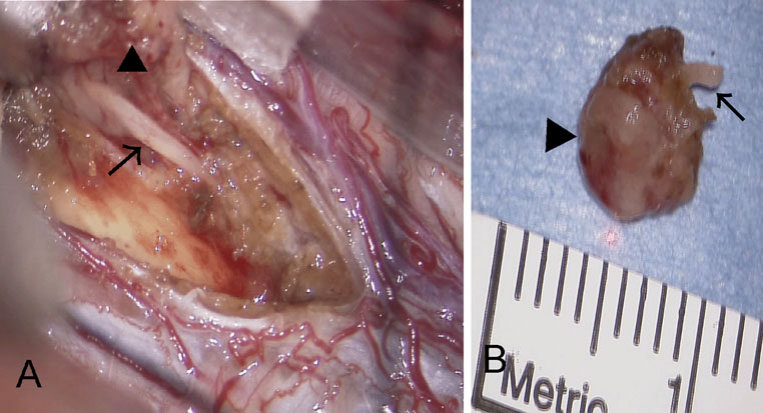
**a** Intraoperative photograph showing whitish string (*arrow*) passing rostrocaudally in the spinal cord and adhering to the ventral side of the spinal tumor (*arrowhead*). **b** Photograph of the resected spinal tumor (*arrowhead*) with whitish string (*arrow*)

The patient underwent surgery in the prone position. A C5–C7 laminectomy was performed to expose the lesion. After dural opening, we found spinal cord swelling. A midline myelotomy was performed and a grayish tumor immediately appeared. The tumor was sharply circumscribed and could be easily resected. When we dissected the ventral side of the tumor near the anterior median sulcus, a whitish string resembling an aberrant nerve root appeared with adhesion to the tumor (Fig. 2a). It was clear that the whitish string was intramedullary, not an anterior root of the spinal nerve. After cutting the whitish string above and below the tumor, we finally resected the tumor en bloc with part of the whitish string (Fig. 2b). Continuous somatosensory evoked potentials were monitored during the surgery. No significant alterations were registered throughout the intervention, compared with the preoperative recordings.

## Histological findings

**Fig. 3:**
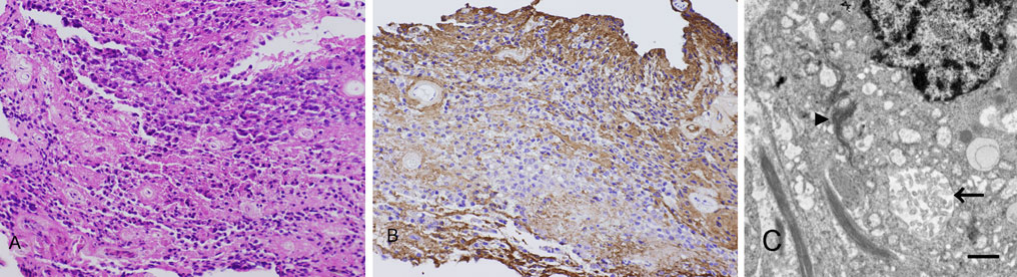
Histological examination of the spinal tumor. **a** Photomicrograph showing perivascular pseudorosettes of small cells with round nuclei and fibrillary elements. HE stain, magnification ×100. **b** Photomicrograph showing reactivity to immunohistochemical staining for glial fibrillary acid protein (GFAP). Magnification ×100. **c** Electron micrograph showing well-developed intermediate junctions (*arrow*), microvilli, and cilia (*arrowhead*). *Bar* indicates 0.5 μm

The specimen was cut and separated into the soft grayish tumor and the tough whitish string. Histopathological examination of the soft grayish tumor revealed perivascular pseudorosettes of small cells with round nuclei and fibrillary elements (Fig. 3a). True ependymal rosettes were not seen. Neither mitosis nor nectotic foci were identified. Immunohistochemical staining showed that the tumor cells were strongly positive for glial fibrillary acid protein (GFAP) (Fig. 3b), vimentin, and S-100 protein, but negative for neurofilament. The Ki-67 labeling index was 0.1 %. Ultrastructual examination found characteristic ependymal features, including well-developed intermediate junctions, microvilli, and cilia (Fig. 3c). On the basis of these findings, the intramedullary tumor was diagnosed as spinal ependymoma (grade 2).

**Fig. 4:**
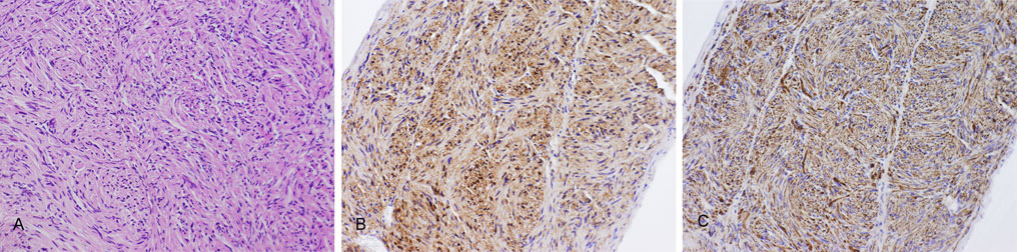
Histological examination of whitish string adhering to the tumor. **a** Photomicrograph showing the proliferation of interlacing large and small bundles of spindle cells with elongated nuclei. HE stain, magnification ×100. **b** The spindle cells were strongly positive for S-100 protein. Magnification ×100. **c** Within the interlacing bundles of spindle cells, immunohistochemical staining showed a striking axonal component that was strongly positive for neurofilament protein. Magnification ×100

Histopathological examination of the tough whitish string revealed the proliferation of interlacing large and small bundles of spindle cells with elongated nuclei (Fig. 4a), which was quite different from ependymoma and similar to schwannoma. Neither mitosis nor nectotic foci were identified. Immunohistochemical staining showed that the spindle cells were strongly positive for vimentin and S-100 protein (Fig. 4b) but negative for GFAP. These findings showed that the spindle cells were Schwann cells. Within the interlacing bundles of Schwann cells, immunohistochemical staining showed a striking axonal component that was strongly positive for neurofilament protein (Fig. 4c). These findings were consistent with an amputation neuroma, which is usually detected in the peripheral nerve; our diagnosis of the tough whitish string associated with a spinal ependymoma was, therefore, intramedullary amputation neuroma. We considered that the intramedullary neuroma in our case might have arisen from intradural tiny peripheral nerves around an anterior spinal artery chronically compressed by the spinal ependymoma.

## Postoperative course

Although her postoperative course was uneventful, the patient did not completely recover from the motor deficit of her hands and legs until 6 months after the operation. Postoperative MR imaging revealed no residual tumor.

## Discussion

Extramedullary neuromas arising from spinal nerve roots in the CNS have been described in the literature [1, 3, 8], and are believed to arise with or without evident history of traumatic injury. Because spinal nerve roots are peripheral nerves, it seems reasonable that extramedullary neuromas occur in spinal canals. The reasons intramedullary neuromas arise have been discussed elsewhere [2, 4, 5, 9, 13].

Intramedullary neuromas might arise from errant axonal migration during embryogenesis [17], and can sometimes be traced to the anterior or posterior roots [18]; usually these hypotheses are impossible, however. Generally, one possible origin of intramedullary neuromas seems to be proliferation of small intramedullary perivascular nerve twigs in an ostensibly normal spinal cord [2, 4, 5, 9, 13]. According to previous reports on spinal cords at autopsy [2, 7, 10, 13], lightly myelinated fibers accompany branches of the anterior spinal artery in the anterior medial sulcus and enter the spinal cord. Thereafter, these tiny peripheral nerves accompanying vessels travel rostrocaudally in the anterior gray commissure near the central canal, and are called aberrant peripheral nerves [2, 7, 10, 13].

As far as we know, only one report has described amputation neuromas associated with spinal ependymomas, and 5 of these cases were microscopic [12]. They also examined 6 normal spinal cords obtained postmortem to visualize the presence of perivascular nerve twigs and incidental neuromas in the spinal cord. In three cases they found microneuromas in the proximity of penetrating arteries in the anterior median sulcus and along small vessels passing rostrocaudally in the gray matter adjacent to the central canal remnants. In addition, in all 3 there were multiple peripheral nerve fibers in the anterior subarachnoid space around the anterior spinal artery. A few were also present in the dorsal subarachnoid space. They speculate in their report that it is reasonable that intramedullary tumors, for example ependymomas, chronically compress and injure these peripheral nerves near the subarachnoid space of the anterior median sulcus and gradually induce amputation intramedullary neuromas.

To the best of our knowledge, no reports have shown operative findings of intramedullary macroneuromas associated with spinal ependymomas. This is the first report with images showing operative findings of the intramedullary neuroma. Our operative findings were that the intramedullary neuroma existed on the ventral side of a ependymoma and passed rostrocaudally, probably close to the anterior median sulcus. These findings seem to support the hypothesis that intramedullary amputation neuromas might derive from intramedullary peripheral nerve twigs around the anterior median sulcus [12]. In our case, the patient had a long history of neurological deficit caused by a spinal ependymoma, which had, perhaps, pressed and injured the peripheral nerves around the anterior median sulcus for a long time; we, therefore, speculate that the intramedullary amputation microneuroma might have grown to become a macroneuroma that could be recognized during surgery.

During surgery, we found a whitish string in spinal cord passing rostrocaudally and attached to the soft tumor; we could not, however, understand what it was. Although histopathological examination enabled easy diagnosis of the primary spinal tumor as spinal ependymoma, another specimen attached to the spinal tumor, part of the whitish string, had features quite different from those of ependymoma. It comprised a proliferation of interlacing bundles of spindle cells with elongated nuclei. At first we believed this specimen was a small schwannoma coexisting with an ependymoma. Immunohistochemical examination, especially immunoreactivity for neurofilament protein, finally led to the exact histological diagnosis of the whitish string in the spinal cord as a neuroma.

Intramedullary neuromas might occur not only with ependymoma but also with other slow-growing tumors, for example subependymomas, pilocytic astrocytomas, and hemangioblastomas. Neurosurgeons should be aware that these tumors might coexist with neuromas, which can be found rarely during surgery. Moreover, if the resected tumor has two different components on microscopic examination, immunohistochemical examination is needed to diagnose an intramedullary “non-neoplastic” neuroma associated with the primary tumor. This should be distinguished from an intramedullary schwannoma, which is a neoplasma without aberrant axons. Pathologists should also be aware of this distinctive intramedullary tissue, so it is not confused with a neoplasm.
